# Construction and Immunogenicity of Virus-Like Particles of Feline Parvovirus from the Tiger

**DOI:** 10.3390/v12030315

**Published:** 2020-03-16

**Authors:** Cuicui Jiao, Hongliang Zhang, Wei Liu, Hongli Jin, Di Liu, Jian Zhao, Na Feng, Chuanmei Zhang, Jing Shi

**Affiliations:** 1 Shandong Collaborative Innovation Center for Development of Veterinary Pharmaceuticals, College of Veterinary Medicine, Qingdao Agricultural University, Qingdao 266109, China; jcc1990512@163.com (C.J.); zhanghongliang001@126.com (H.Z.); 2 Changchun Sino Biotechnology Co., Ltd., Changchun 130012, China; liuwei200314@163.com (W.L.); liudi8680@163.com (D.L.); zhj15948095541@163.com (J.Z.); 3 Key Laboratory of Zoonosis Research, Ministry of Education, College of Veterinary Medicine, Jilin University, Changchun 130062, China; jin8616771@163.com; 4 Key Laboratory of Jilin Province for Zoonosis Prevention and Control, Military Veterinary Research Institute, Academy of Military Medical Sciences, Changchun 130122, China

**Keywords:** feline parvovirus, virus-like particles, VP2 protein, antibodies

## Abstract

Feline panleukopenia, caused by feline parvovirus (FPV), is a highly infectious disease characterized by leucopenia and hemorrhagic gastroenteritis that severely affects the health of large wild Felidae. In this study, tiger FPV virus-like particles (VLPs) were developed using the baculovirus expression system. The VP2 gene from an infected Siberian tiger (*Panthera tigris altaica*) was used as the target gene. The key amino acids of this gene were the same as those of FPV, whereas the 101st amino acid was the same as that of canine parvovirus. Indirect immunofluorescence assay (IFA) results demonstrated that the VP2 protein was successfully expressed. SDS-PAGE and Western blotting (WB) results showed that the target protein band was present at approximately 65 kDa. Electron micrograph analyses indicated that the tiger FPV VLPs were successfully assembled and were morphologically similar to natural parvovirus particles. The hemagglutination (HA) titer of the tiger FPV VLPs was as high as 1:2^18^. The necropsy and tissue sections at the cat injection site suggested that the tiger FPV VLPs vaccine was safe. Antibody production was induced in cats after subcutaneous immunization, with a >1:2^10^ hemagglutination inhibition (HI) titer that persisted for at least 12 months. These results demonstrate that tiger FPV VLPs might provide a vaccine to prevent FPV-associated disease in the tiger.

Feline panleukopenia, caused by feline parvovirus (FPV), is an acute and highly contagious disease [1]. It clinically manifests as vomiting, high fever, leukopenia and enteritis in infected animals [2]. FPV has been known to induce disease in cats since the 1920s [3]. Since its discovery, FPV has been isolated from cats, raccoons, monkeys, and many different wild and captive carnivores [4,5]. FPV is very stable in the environment, with infectivity persisting for up to one year within contaminated material [6]. Consequently, FPV has a high risk of transmission, posing a great threat to rare wild animals. In addition, FPV is prevalent in the Chinese Siberian tiger (*Panthera tigris altaica*) population [7,8]. On June 6th 2016, an outbreak of fatal FPV infection among captive Siberian tigers in Zhengzhou Zoo in central China was reported [7]. An FPV strain was isolated and identified from a captive Siberian tiger in a wildlife park in the Jilin Province, China [8]. Wild Siberian tigers from the Russian Far East had a 68% FPV antibody-positivity rate [9]. Vaccination is an effective means to prevent FPV infection. Currently, attenuated vaccines and inactivated vaccines are approved for use against FPV in cats. Attenuated vaccines have been recommended for wildlife use in North American countries, Europe and Asia [10]. A live-attenuated vaccine (Virbac S.A., Carros 06511, France), which combines feline calicivirus (FCV), feline herpesvirus (FHV) and FPV strains, is used to prevent disease in tigers [11]. However, it has been reported that attenuated FPV vaccines with a risk of virulence reversion can cause cerebellar dysplasia in cat fetuses [12]. The antibody level produced by inactivated vaccines is low, the antibody duration is short, and there is a risk of incomplete inactivation. Furthermore, the safety of using inactivated vaccines in tigers remains unknown. Therefore, the development of a novel vaccine for feline parvovirus disease in tigers will provide material reserves and new products for immune protection against important diseases in tigers.

FPV, a single-stranded DNA virus, belongs to the family *Parvoviridae*, subfamily *Parvovirinae*, genus *Protoparvovirus* [13]. The virions have a symmetrical icosahedral structure [14]. The FPV genome contains two open reading frames encoding nonstructural proteins (NS1 and NS2) and structural proteins (VP1 and VP2). The VP2 protein contains the major epitopes that stimulate the production of neutralizing antibodies [15], and mutations in key amino acid sites of the VP2 protein can affect its antigenic characteristics and host range [16,17].

Virus-like particles (VLPs) are composed of multiple copies of one or more recombinant viral structural proteins and are spontaneously assembled into particles without the viral genome [18]. Due to being replication-incompetent and producing nonpathogenic effects, VLPs have been widely used in studies of human and veterinary candidate vaccines [19]. In this study, the parvovirus VP2 gene was identified from a dead Siberian tiger in a wildlife park in the Jilin Province, China [8]. The tiger had symptoms such as diarrhea and vomiting before it died. The VP2 sequence was analyzed and kindly provided by Dr. Wang [8]. We used the baculovirus expression system [20] to produce the VP2 protein of tiger FPV. The VP2 protein can be assembled into VLPs which resemble the natural virus in size and shape. Meanwhile, tigers and cats both belong to the family Felidae. Due to the rarity and importance of the tiger, cats were selected to conduct immunization experiments of the tiger FPV VLPs vaccine. Herein, the tiger FPV VLPs vaccine stimulated cats to produce hemagglutination inhibition (HI) antibody, providing a preliminary basis and technical support for the preparation of new vaccines against tiger (*Panthera tigris altaica*) feline parvovirus disease.

First, the amino acid sites of the parvovirus VP2 gene from a tiger were analyzed. The results showed that most of the amino acid sites were consistent with those of the FPV reference sequence, whereas the 101st amino acid was consistent with that of the canine parvovirus (CPV) reference sequence (Table 1). Therefore, the parvovirus VP2 gene from the tiger was named tiger FPV VP2.

Next, we optimized the codons of tiger FPV VP2 for *Spodoptera frugiperda* 9 (Sf9) cells according to codon preference (shown in Supplementary Materials, Sequence 1). Sequence optimization and synthesis were completed by Shanghai Biological Engineering Co., Ltd. The VP2 gene was amplified using the primers shown in Table 2 and cloned into the pFastBac Dual vector (Invitrogen, Carlsbad, CA, USA). The pFBD-dVP2 vector, a donor plasmid containing two copies of the VP2 gene, was successfully constructed. The pFBD-dVP2 donor plasmid was transformed into *E. coli* DH10Bac competent cells (Invitrogen, Carlsbad, CA, USA) to obtain the rBacmid-dVP2 recombinant bacmid.

The rBacmid-dVP2 was transfected into Sf9 cells and cultured in six-well plates at approximately 80% confluence with Cellfectin II Reagent Transfection Reagent (Invitrogen, Carlsbad, CA, USA) according to the manufacturer’s instructions, and the cells were then cultured in sf-900 II SFM medium (Gibco, Grand island, NY, USA) at 27 °C. It took approximately 96 h to reach an 80% cytopathogenic effect (CPE). The supernatant was collected to obtain recombinant baculovirus, called rpFBD-dVP2 which was passage 1 (P1). The genome was extracted and determined to be correct by PCR. The baculovirus stock titer was measured using a Baculovirus Rapid Titer Kit (TaKaRa, Tokyo, Japan) according to the manufacturer’s instructions. RpFBD-dVP2 was continuously passaged to P4 at a multiplicity of infection (MOI) of 0.5, and obvious CPE was observed at 48 h, with large, rounded and shed cells (Figure 1A).

The expression of VP2 protein was verified by an indirect immunofluorescence assay (IFA). Sf9 cells were infected with rpFBD-dVP2 at a multiplicity of infection (MOI) of 0.5 and fixed after two days. A monoclonal antibody against CPV (clone 8H7, HyTest Ltd., Turku, Finland) was used as a primary antibody (1:200), and fluorescein isothiocyanate (FITC)-labeled sheep anti-mouse IgG (Solarbio, Beijing, China) was used as the secondary antibody (1:200). The IFA results showed obvious green fluorescence (Figure 1Ba), and no specific fluorescence was observed in normal Sf9 cells (Figure 1Bb). This result indicated that VP2 protein was expressed in Sf9 cells and could be specifically recognized by the VP2 monoclonal antibody.

Sf9 suspension cells were cultured in triangular flasks with sf-900 II SFM (Gibco, Grand island, NY, USA) at a speed of 120 rpm. To assess the expression of VP2 protein, Sf9 suspension cells were harvested 4 days after infection. The mixture was separated into cells and supernatant at 1751× *g* for 30 min. The cells were lysed with filtered 25 mM NaHCO_3_, after which the supernatant was harvested by centrifugation. The samples were characterized by SDS-PAGE, and a clear band was observed at 65 kDa (Figure 1C). The results showed that VP2 protein was successfully expressed in Sf9 cells and released into the supernatant after simple cell lysis. A Western blotting (WB) assay was carried out to verify whether the 65 kDa band was the target protein. Samples from SDS-PAGE were transferred to a nitrocellulose (NC) membrane (GE Healthcare, Dassel, Germany). In the WB assay, as the primary antibody, the monoclonal antibody against CPV (clone 8H7, Hytest, Turku, Finland) at a 1:500 dilution was incubated at room temperature (RT) for 1.5 h. Then, the horseradish peroxidase (HRP)-labeled sheep anti-mouse IgG at a 1:5000 dilution was incubated at RT for 1 h. As shown in Figure 1D, the WB analysis results showed that the target protein specifically bound the monoclonal antibody against parvovirus; a specific band was seen at the molecular weight of approximately 65 kDa in lane 2, whereas no specific band was seen in the control sample. These results indicated that the expressed VP2 protein had the capacity to elicit a specific antibody response.

HA tests were carried out as previously described to detect VP2 expression levels [22]. The HA titer was determined by the highest dilution of tiger FPV VLPs that caused pig erythrocytes agglutination. The results in Figure 2A showed that the HA titer of tiger FPV VLPs reached 1:2^18^. High yields of tiger FPV VLPs could be produced due to the high HA titer [23]. To inspect their morphology, the tiger FPV VLPs were observed by electron microscopy. The results showed that the tiger FPV VLPs were successfully assembled with a diameter of ~22 nm; their morphology was similar to that of natural parvovirus particles (Figure 2B).

The supernatant containing VLPs after cell lysis was used to prepare the vaccine. Tiger FPV VLPs were emulsified with Gel 02 adjuvant (Seppic, Paris, France) at a volume ratio of 7:1. To determine whether the vaccine was safe for cats, an overdose vaccination test was carried out on cats. Two 10-week old cats were subcutaneously immunized in the neck with 2 mL of tiger FPV VLPs vaccine and given an immunization boost with the same dose four weeks later. The feces production, behavior, and food and water consumption were observed continuously for 14 days after the last immunization. Fourteen days later, the immunized cats were euthanized. The injection site was dissected to observe whether there were side effects, such as nodules, and the tissue was sectioned to detect pathological changes. No anomalies in the daily observation parameters were observed in cats immunized with tiger FPV VLPs vaccine. There were no sarcomas in the injection site after necropsy (Figure 3Aa), which indicated that the absorption of the vaccine was good and that the tissue sections at the injection site were not abnormal (Figure 3Ab). These results demonstrated that the tiger FPV VLPs vaccine was safe for cats.

To detect the immune effects, two 10-week old cats were subcutaneously immunized with 1 mL of tiger FPV VLPs vaccine, and one cat was given PBS as a control. Identical vaccinations were then repeated at four weeks after the primary immunization. Blood samples were collected at 2, 4, 5, 6, and 7 weeks and at 2, 3, 4, 5, 6, 7, 8, 9, 10, and 12 months after the primary immunization. Antibody levels of tiger FPV in the sera were measured by HI assay using pig erythrocytes, and the assay was performed as described previously [24]. The results, shown in Figure 3B, indicated that the anti-FPV HI titer was detected in the immunized cats two weeks after primary immunization, with a titer of approximately 1:2^10^. After boost immunization, the HI titer was essentially stable at 1:2^10^. The anti-FPV HI titer was still detected 12 months after the primary immunization, and the antibody titer was not less than 1:2^10^ (Figure 3B). The second batch of immunization was conducted in other independent tests, which are still under observation. The tendency of HI titer after immunization was the same as above, with HI titers of 1:2^9^–1:2^10^ six months after immunization (Figure S1). These results showed that in cats, tiger FPV VLPs effectively stimulated the immune response and induced the production of anti-FPV antibodies, which had a good immune effect.

VP2 is the main component of the capsid protein of FPV and determines its antigenicity [25]. The amino acid sites of the VP2 protein from tiger FPV were analyzed in this article. The 101st amino acid was consistent with that of CPV, and the rest of the amino acid variations in the VP2 protein [21] were consistent with those of FPV. It has been speculated that the strain might be the product of a recombination event or mutation in response to selection pressure. Therefore, the parvovirus VP2 gene from a tiger was selected to generate a vaccine for tiger parvovirus, which is more representative than other strains and provides a method to prevent and treat tiger parvovirus disease.

In this study, tiger FPV VLPs were developed using the baculovirus expression system, and the expressed protein was identified, confirming that VP2 protein was expressed in vitro and could self-assemble into VLPs. The recombinant baculovirus infected Sf9 cells, cultured in suspension, could express the VP2 protein at high yields and with a high HA titer, indicating their suitability for production on a large scale. VLPs are generally safer than inactivated and attenuated vaccines due to their lack of viral nucleic acids and infectivity. In addition, Gel 02 adjuvant, which is a water-soluble adjuvant, was used in this study. There were no sarcomas and site effects in cats immunized with the tiger FPV VLPs vaccine emulsified with Gel 02 adjuvant. Historically, HI is considered the gold standard for FPV antibody detection [26]. Generally, a HI titer of ≥1:40 is considered protective against FPV [27]. The highest HI titer in the sera of the cats in this experiment reached 1:2^13^, and the HI titer remained stable at 1:2^10^ after 12 months. The successful preparation of tiger FPV VLPs provides a new approach for the prevention and control of tiger parvovirus disease and technical support for the prevention of rare wild animal viral diseases. Due to the rarity and importance of tigers, relevant institutions are particularly cautious about the inoculation of tigers. Therefore, cats of the same family as tigers were selected for the evaluation of the vaccine immunity effect in this study, with the aim of developing a new type of safe and effective vaccine against feline parvovirus disease in tigers and laying a foundation for the prevention, control and treatment of feline parvovirus disease. In the subsequent application, the tiger FPV VLPs vaccine can be used as a single vaccine or a multiple vaccine combined with other vaccines for routine immunizations of tigers in zoos.

In this study, animal experimentation was handled in compliance with the guidelines and protocols of the Welfare and Ethics of Laboratory Animals of China. The animal studies were approved by the Animal Welfare and Ethics Committee of Changchun Sino Biotechnology Co., Ltd.

## Figures and Tables

**Figure 1:**
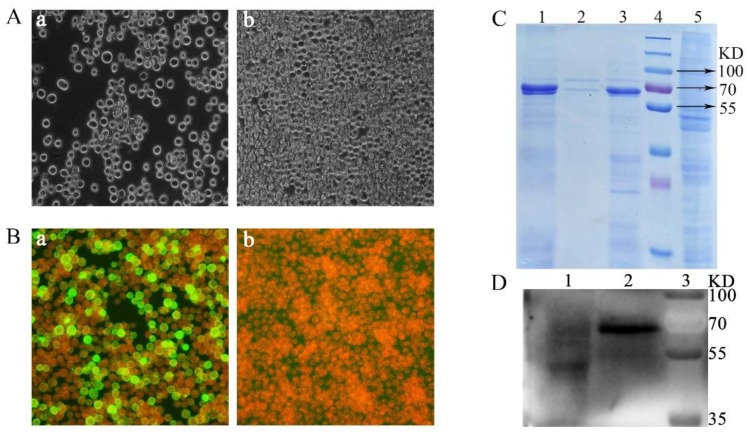
Recombinant baculovirus acquisition and the identification of VP2 protein expressed in Sf9 cells. (**A**) Cytopathogenic effect (CPE) of the recombinant rpFBD-dVP2 baculovirus (magnification, 200×). (Aa) Sf9 cells infected with rpFBD-dVP2 displayed typical CPE 48 h after infection, with large, rounded and shed cells, and (Ab) untreated Sf9 cells remained normal. (**B**) Indirect immune fluorescence antibody staining of VP2 protein in Sf9 cells infected with rpFBD-dVP2 (magnification, 200×). (Ba) Obvious green fluorescence was observed in Sf9 cells infected with rpFBD-dVP2, and (Bb) untreated Sf9 cells showed no fluorescence. (**C**) SDS-PAGE analysis of VP2 protein expression. Lane 1, untreated mixture; lane 2, supernatant after centrifugation; lane 3, supernatant after sodium bicarbonate treatment; lane 4, prestained protein markers; lane 5, cells infected with control baculovirus. (**D**) WB analysis of VP2 protein expression. Lane 1, cells infected with control baculovirus; lane 2, cells infected with recombinant baculovirus; lane 3, prestained protein markers.

**Figure 2:**
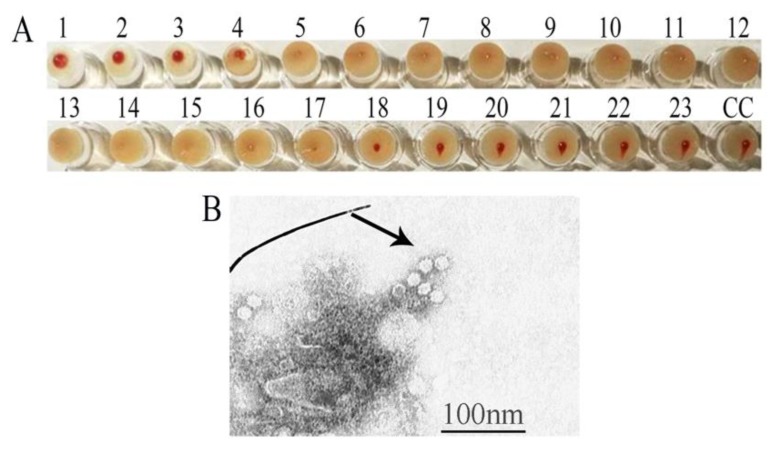
Determination of HA titer and observation of tiger FPV VLPs morphology. (**A**) HA activity of tiger FPV VLPs. (**B**) Electron micrograph analysis of tiger FPV VLPs. The arrow indicates VLPs, and the bar represents 100 nm.

**Figure 3:**
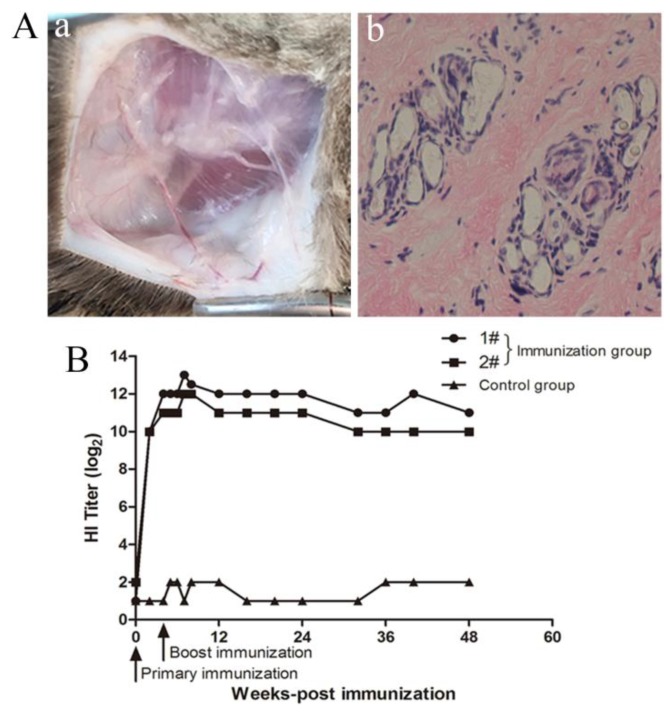
Safety and immunogenicity assay of tiger FPV VLPs. (**A**) Injection site necropsy and tissue section results. (Aa) There were no sarcomas in the injection site after necropsy. (Ab) The tissue sections were normal. (**B**) The immunogenicity of tiger FPV VLPs in cats was evaluated by HI assay after immunization. Cats were immunized twice via subcutaneous injection at four-week intervals. The immunization group was immunized with tiger FPV VLPs mixed with Gel 02 adjuvant. The control group was immunized with PBS.

**Table 1:** Amino acid variations in the VP2 protein.

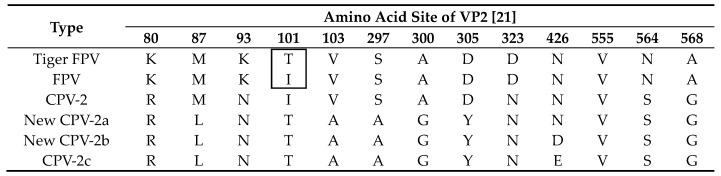

The box shows the difference between Tiger FPV and FPV at 101st amino acid.

**Table 2:** Primer sequences.

Primer	Primer Sequence (5′-3′)	Product Length
PH-VP2-F	TAT**GCGGCCGC**ATGTCCGACGGTGCTGT (*Not* I)	1755 bp
PH-VP2-R	TAT**AAGCTT**TTAGTACAGCTTACGAGGA (*Hind* III)	
P10-VP2-F	TAT**CTCGAG**ATGTCCGACGGTGCTGTGC (*Xho* I)	1755 bp
P10-VP2-R	TAT**GGTACC**TTAGTACAGCTTACGAGGAG (*Kpn* I)	

The bold characters are the sequences of restriction enzyme sites.

## Supplementary Materials

The following are available online at https://www.mdpi.com/1999-4915/12/3/315/s1, Figure S1: The immunogenicity of tiger FPV VLPs in cats was evaluated by HI assay, Sequence S1: The codon-optimized tiger parvovirus VP2 sequences.
