# Supplementary material for: Construction and Immunogenicity of Virus-Like Particles of Feline Parvovirus from the Tiger
Source: Viruses. 2020 Mar 16;12(3):315. doi: 10.3390/v12030315 (PMC7150758; doi:10.3390/v12030315)
Supplement: Supplementary file 1 [file viruses-12-00315-s001.pdf]

Supplementary Materials

Sequence S1. The codon-optimized tiger parvovirus VP2 sequences

ATGAGTGATGGAGCAGTTCAACCAGACGGTGGTCAACCTGCTGTCAGAAATGAAAGAGCTGCAGGATCTG
GGAACGGATCTGGAGGCGGGGGTGGTGGTGGTTCTGGGGATGTGGGGATTTCTACGGGTACTTTCAATAAT
CAGACGGAATTTAAATTTTTGGAAAACGGATGGGTGGAAATCACAGCAAACTCAAGCAGACTTGTACATTTA
AATATGCCAGAAAGTGAAAATTATAAAAGAGTAGTTGTAAATAATATGGATAAAACTGCAGTTAAAGGAAACA
TGGCTTTAGATGATACTCATGTACAAATTGTAACACCTTGGTCATTGGTTGATGCAAATGCTTGGGGAGTTTG
GTTTAATCCAGGAGATTGGCAACTAATTGTTAATACTATGAGTGAGTTGCATTTAGTTAGTTTTGAACAAGAA
ATTTTTAATGTTGTTTTAAAGACTGTTTCAGAATCTGCTACTCAGCCACCAACTAAAGTTTATAATAATGATTTA
ACTGCATCATTGATGGTTGCATTAGATAGTAATAATACTATGCCATTTACTCCAGCAGCTATGAGATCTGAGAC
ATTGGGTTTTTATCCATGGAAACCAACCATACCAACTCCATGGAGATATTATTTTCAATGGGATAGAACATTAA
TACCATCTCATACTGGAACTAGTGGCACACCAACAAATGTATATCATGGTACAGATCCAGATGATGTTCAATTT
TATACTATTGAAAATTCTGTGCCAGTACACTTACTAAGAACAGGTGATGAATTCGCTACAGGAACATTTTTTTT
TGATTGTAAACCATGTAGACTAACACATACATGGCAAACAAATAGAGCATTGGGCTTACCACCATTTCTAAATT
CTTTGCCTCAATCTGAAGGAGCTACTAACTTTGGTGATATAGGAGTTCAACAAGATAAAAGACGTGGTGTAA
CTCAAATGGGAAATACAGACTATATTACTGAAGCTACTATTATGAGACCAGCTGAGGTTGGTTATAGTGCACC
ATATTATTCTTTTGAAGCATCTACACAAGGGCCATTTAAAACACCTATTGCAGCAGGACGGGGGGGAGCGCA
AACAGATGAAAATCAAGCAGCAGATGGTGATCCAAGATATGCATTTGGTAGACAGCATGGTCAAAAAACTAC
TACAACAGGAGAAACACCCGAGAGATTTACATATATAGCACATCAAGATACAGGAAGATATCCAGAAGGAGA
TTGGATTCAAAATATTAACTTTAACCTTCCTGTAACAAATGATAATGTATTGCTACCAACAGATCCAATTGGAG
GTAAAACAGGAATTAACTATACTAATATATTTAATACTTATGGTCCTTTAACTGCATTAAATAATGTACCACCAGT
TTATCCAAATGGTCAAATTTGGGATAAAGAATTTGATACTGACTTAAAACCAAGACTTCATGTAAATGCACCAT
TTGTTTGTCAAAATAATTGTCCTGGTCAATTATTTGTAAAAGTTGCGCCTAATTTAACAAATGAATATGATCCTG
ATGCATCTGCTAATATGTCAAGAATTGTGACTTACTCAGATTTTTGGTGGAAAGGTAAATTAGTATTTAAAGCT
AAACTAAGAGCATCTCATACTTGGAATCCAATTCAACAAATGAGTATTAATGTAGATAACCAATTTAACTATGTA
CCAAATAATATTGGAGCTATGAAAATTGTATATGAAAAATCTCAACTAGCACCTAGAAAATTATATTAA

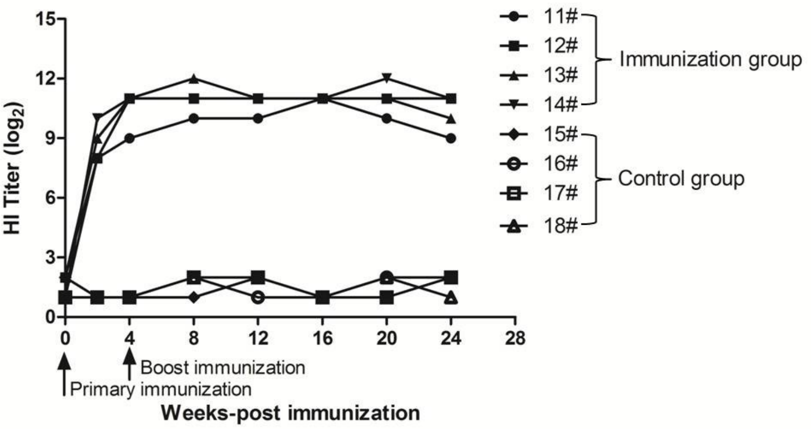

Figure S1. The immunogenicity of tiger FPV VLPs in cats was evaluated by HI assay

Four Cats were immunized twice via subcutaneous injection at four-week intervals. The immunization group was immunized with tiger FPV VLPs mixed with Gel 02 adjuvant. The control group was injected with PBS.
